# Data on Ocrelizumab Treatment Collected by MS Patients in Germany Using Brisa App

**DOI:** 10.3390/jpm14040409

**Published:** 2024-04-12

**Authors:** Steffeni Papukchieva, Maria Kahn, Markus Eberl, Benjamin Friedrich, Natalie Joschko, Tjalf Ziemssen

**Affiliations:** 1 Temedica GmbH, 80687 Munich, Germany; steffeni.papukchieva@temedica.com (S.P.);; 2 Roche Pharma AG, 79639 Grenzach-Wyhlen, Germany; 3 Center of Clinical Neuroscience, Department of Neurology, University Clinic Carl Gustav Carus & Dresden University of Technology, 01307 Dresden, Germany

**Keywords:** multiple sclerosis, Brisa, patient reported outcomes, digital health

## Abstract

Background: With a rising number of multiple sclerosis (MS) cases and increasing pressure on health systems, digital companion apps like Brisa, designed specifically for people with MS, can play an important role in the patient journey. These apps enable the collection of real-time longitudinal data that are critical to our understanding of the pathophysiology and progression of MS. Methods: This retrospective, descriptive analysis consists of data from Brisa users who registered between 6 August 2021 and 8 September 2022. Of the unique users, 37.7% (*n* = 1593) fulfilled the inclusion criteria including information about medication and demographics and tracked one or more symptoms and/or patient-reported outcomes. Users were classified as moderate-efficacy treatment users, high-efficacy treatment users and ocrelizumab users, and the reporting frequency and scores of symptoms and patient-reported outcomes were analyzed. Results: The largest cohort of Brisa users (405) reported treatment with ocrelizumab and were mostly diagnosed 2–5 years before the survey. The most reported MS symptoms were similar between OUs (ocrelizumab users), HETUs (high-efficacy treatment users) and METUs (moderate-efficacy treatment users). OUs on average reported symptoms and answered questionnaires more frequently. Baseline scores between HETUs and OUs were similar, whereas baseline scores of METUs were slightly lower in comparison. In a further analysis of OUs, disability scores increased with age; users aged 26–45 years had higher pain scores than 18–25-year-olds. No significant differences were found in quality of life, bowel control and vision between age groups. Conclusion: These findings show that the characteristics of the Brisa cohort are similar to the results of other studies and registries and can provide a representative overview of everyday disease management. Thereby, these results can bridge the gap between clinical research and real patient experience, but they also raise new questions, such as how often the hard-and-early therapy approach is already used and whether baseline characteristics and reasons for choosing a particular treatment contribute to the different outcomes over time. Answering these questions requires further research and analysis.

## 1. Introduction

In recent years, more and more medical device applications have entered the healthcare landscape, therefore placing decision-making power directly into patients’ hands. Especially in chronic diseases like multiple sclerosis (MS), the continuous collection of patient-generated data is crucial to understanding the very individual path of a patient’s disease and treatment journey. 

Currently, it is not uncommon for people diagnosed with MS to see their doctor only two to three times a year [1]. These long periods between medical check-ups can be perceived as a gap in patient care. As the number of MS cases increases worldwide [2], digital companion apps may be an option for many patients [3]. Due to the individualistic nature of MS, the collection of real-time data on a longitudinal basis—along with a variety of digital biomarkers—afforded by such apps is becoming increasingly crucial to our understanding of this disease and its pathophysiology and progression [4,5]. Brisa is an example of an MS companion that allows the daily tracking of the range of symptoms MS patients suffer from. Monitoring symptoms could be crucial for detecting relapses and identifying progression, but it also empowers the patients to be part of the therapy decision-making process and exchange their journey in a community. Also, Ziemssen et al. have already shown the importance of a physician-completed tool for supporting physician–patient interaction in assessing signs of disease progression and uncovered the need for supporting tools [6]. 

The collection of patient-centered data in chronic diseases such as MS is becoming increasingly important. Understanding how patients experience their symptoms and how these symptoms affect their daily lives is essential to improve patient care and treatment outcomes [7]. Additionally, the possibility of close symptom monitoring by patients themselves could make a crucial contribution to knowledge about and identification of signs of disease progression, as well as empowering patients to steer towards a personalized therapy approach. 

The optimal disease management for MS is being revised with an increasing emphasis on personalized treatment approaches [8]. Over the past 25 years, the treatment approach and disease management have changed significantly [9]. While patients have traditionally been treated in an escalating manner starting from lower efficacy treatment and moving to higher efficacy treatment only after ongoing treatment fails [10], increasing evidence emphasizes the importance of an early intervention following diagnosis accompanied by optimization of treatment for each patient individually [11,12]. Variations in symptoms can occur from person to person depending on the severity of the neuronal damage, and although high-efficacy therapies are available, some patients still suffer from relapses and subclinical disease activity. Relapses and disease progression can take place in different functional systems through a wide range of symptoms that include fatigue, impaired motor function, spasticity, pain, gait disturbance, speech problems and cognitive impairment [4,13,14]. These symptoms can have a negative impact on individuals both physically and psychologically, with the progression of this disease leading to difficulties in performing everyday tasks due to impaired motor skills and affecting social life and the ability to live independently [15]. Especially in a disease termed ‘disease of a thousand faces’, it is crucial to gain an understanding of all relevant symptoms not only to assess signs of disease activity and progression, but also to improve patients’ disease management.

While progress has been made in terms of developing new treatments due to a more comprehensive understanding of the course and pathogenesis of MS [14], MS is still considered to be an incurable disease [4]. A variety of medications, with moderate to high efficacy, are available for the treatment of MS, particularly the relapsing–remitting form (RRMS) [16]. For the primary progressive form of MS (PPMS), ocrelizumab is currently the only approved medication [9,10]. Therefore, ocrelizumab, approved for both RMS and PPMS, occupies a special position in the treatment landscape and has already been proven to fulfill the safety profile, treatment persistence and adherence of clinical trials in a real-world MS population [17]. The current study focused on characteristics, symptoms and PRO reporting and the corresponding scores of Brisa users who are treated with medium- and high-efficacy drugs, with an individual assessment of ocrelizumab users representing the biggest treatment cohort of Brisa users. 

The aim of this work is to better understand the Brisa users treated with high- and medium-efficacy therapies and to assess differences reflected in symptom and PRO reporting and scores.

## 2. Methods

### 2.1. Ethical Approval

Since the data were aggregated and analyzed retrospectively and Brisa users consented to the use of their data, ethical approval is not applicable.

### 2.2. Data Source

Brisa (version 2.1.0) is a smartphone application available for Android and iOS devices intended to support MS patients in their day-to-day lives by offering guidance and advice in a variety of areas. The data source and collection were described previously [18,19] 

### 2.3. Inclusion Criteria of Study Cohort

This retrospective, descriptive analysis consists of data from Brisa users who registered between 6 August 2021 and 8 September 2022. Of all registered Brisa users (*n* = 6092), 69.4% (*n* = 4228) gave consent to the use of their data for scientific purposes. In the timeframe of analysis, on average, 65 users actively used the app (answered a PRO and/or reported a symptom) on a daily basis, 164 weekly and 412 monthly. Of the unique users, 37.7% (*n* = 1593) fulfilled the inclusion criteria listed below and were therefore included in our analysis. The inclusion criteria were age 18–80 years, gender reported, MS type reported, year of diagnosis reported, medication reported, consent for health data usage for scientific purposes, answered at least one questionnaire completely and reported at least one symptom.

### 2.4. Data Processing and Analysis

Data processing and analysis were performed using Python (version 3.9). To study the demographic characteristics of Brisa users/patients such as gender, age, type of MS, time since MS diagnosis and medications, onboarding information was examined. For each parameter analyzed, only those who responded and provided details for the corresponding parameter were considered. Users with skipped (categorized as ‘unknown’) or invalid entries were excluded. Additional inclusion criteria specific for each parameter under investigation and their classification were as follows:

Age was calculated using the year of birth. For all age-related analyses, users between the ages of 18 and 85 were considered. They were further classified into 5 subgroups based on age: 18–25, 26–35, 36–45, 46–55, >55 years.

To study gender-based age distribution, users who reported both parameters were included. This applies to all cases throughout the analysis where two or more parameters were involved, unless mentioned otherwise.

Time since diagnosis was computed using the year of diagnosis. All entries up to 30 years since diagnosis were considered for analysis. Based on the years since diagnosis, users were further grouped into 5 categories: 0–1 year, 2–5 years, 6–10 years, 11–20 years, and 21–30 years.

All patients with known medication entries were included.

Based on recent publications [20], users were grouped into moderate-efficacy treatment users (METUs), high-efficacy treatment users (HETUs) and ocrelizumab users (OUs) (Table 1)

To identify the symptoms that predominantly affected our study cohort, users who tracked at least 1 symptom once after onboarding were considered.

### 2.5. Statistical Methodology

Statistical analysis was performed using Graphpad (version 10.0). To calculate significant differences in base scores, we ran a multiple-comparison Kruskal–Wallis test correcting for *p*-values using Dunn’s test. *p*-values < 0.05 were considered significant.

## 3. Results

We characterized our cohort by studying their demographic features such as gender, age, MS type, year of diagnosis and medications used. In addition, we examined the symptoms and PROs users on different medication classes are concerned about most and evaluated their scores with an in-depth analysis of the ocrelizumab users (OUs) since they represented the biggest cohort of patients using a high-efficacy medication.

### 3.1. Demographic Characteristics of Users

Data from 1593 Brisa users were available for analysis. As shown in Table 1, 405 Brisa users entered the usage of ocrelizumab as their treatment, whereas 1188 Brisa users stated a medication other than ocrelizumab. A detailed distribution of medications can be seen in Supplementary Figure S1. The distribution regarding gender and MS type (Table 2) is according to the normal gender and MS-type distribution. Table 3 and Table 4 show specifically the distribution of ocrelizumab users. While users diagnosed with RRMS/SPMS decrease with age, users diagnosed with PPMS increase with age. Most ocrelizumab users (OUs) were diagnosed 2–5 years before the survey. 

### 3.2. Symptoms Reported by the Different Treatment Groups

**Figure 1:**
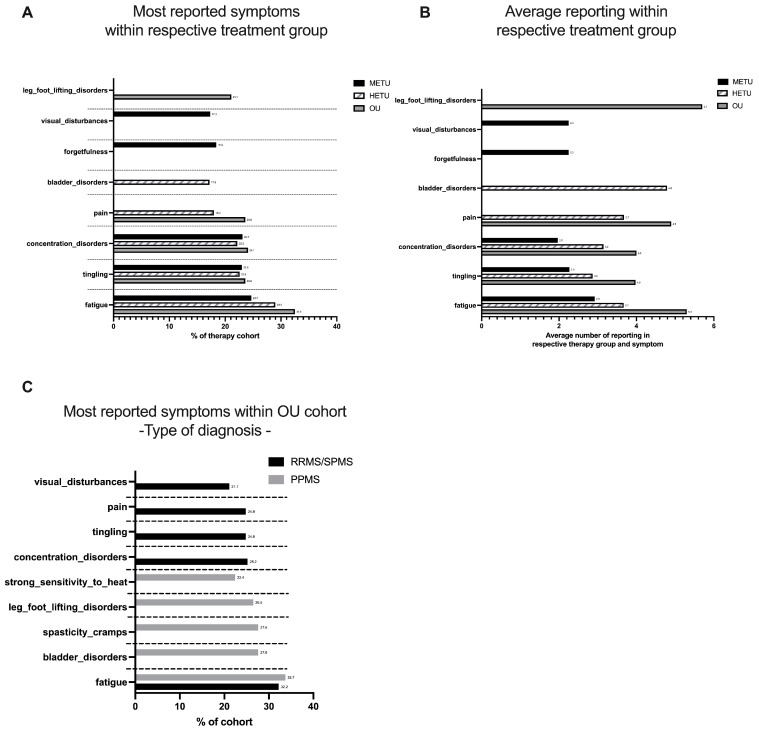
Symptoms that concern Brisa users most. (**A**) Brisa users were grouped into three categories based on the medications they use. Top 5 most reported symptoms of each respective treatment group are depicted for ‘moderate-efficacy treatment users’ (METUs), ‘high-efficacy treatment users’ (HETUs) and ‘ocrelizumab users’ (OUs). (**B**) Average reporting. Average number of reported symptoms based on the number of all users that reported the respective symptom at least once in the respective treatment group during the observation period. (**C**) Most reported symptoms of OUs grouped by MS type.

To further understand the symptoms that ocrelizumab users (OUs) are mainly dealing with and how they differ from non-ocrelizumab users, we examined the top five most reported symptoms in each treatment category. While fatigue, tingling and concentration disorder were reported by more than 20% of the users of the different treatment cohorts, moderate-efficacy treatment users (METUs) were the only ones to report visual disturbances and forgetfulness. Bladder disorders were only within the top five reported symptoms in the high-efficacy treatment user (HETU) group, while leg foot lifting disorder was only listed in the ocrelizumab user cohort. Pain was listed in both the HETU and OU cohorts (Figure 1A). Figure 1B shows the average number of times users in the respective treatment group answered the symptom of interest. Overall, OUs reported their top five symptoms more often during the observation period than the HETU and METU cohorts. Leg foot lifting disorder was tracked on average 5.7 times during the observation period, whereas concentration disorder and tingling were tracked least with an average of 4 times. METUs reported their top five symptoms at least with an average of around 2.4 times during the observation period.

The deep dive into OUs shows that users diagnosed with PPMS tracked different top five symptoms (bladder disorder, spasticity/cramps, leg foot lifting disorder and strong sensitivity to heat) compared to users diagnosed with RMS (RRMS/SPMS) (concentration disorder, tingling, pain, visual disturbances). Fatigue was the most tracked symptom in both groups (Figure 1C). A focus on the age groups within the OU cohort revealed that fatigue was mentioned in all age groups, whereas visual disturbances were mentioned in the top five only in the age group 26–35. Age groups 46–55 and >55 tracked leg foot lifting disorders, whereas sensitivity to heat was only represented in the age group 36–45. Depression was one of the top five symptoms reported in the youngest age group (Table 5). 

### 3.3. Top Five Completed Patient Reported Outcomes (PROs)

**Figure 2:**
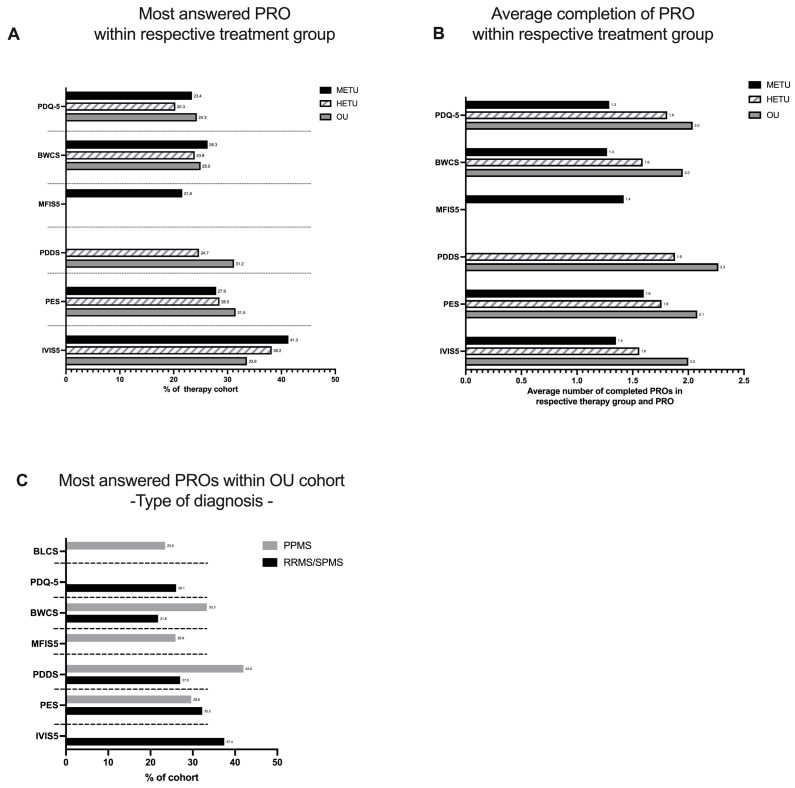
PROs that concern Brisa users most. (A) Brisa users were grouped into three categories based on the medications they use. Top 5 most completed PROs of each respective treatment group are depicted for ‘moderate efficacy treatment users’ (METUs), ‘high efficacy treatment users’ (HETUs) and ‘ocrelizumab users’ (OUs). (**B**) Average reporting. Average number of completed PROs based on the number of all users that completed the respective PRO at least once in the respective treatment group during the observation period. (**C**) Most completed PRO of OU grouped by MS type.

Similarly to daily reported symptoms, we also analyzed the completion of PROs to gain better insights into which PROs are mainly reported by METUs, HETUs and OUs. As shown in Figure 2A, vision (IVIS5) was reported by more than 30% of the respective treatment cohort. Pain (PES), (bowel control) BWCS and cognition (PDQ-5) were among the top five reported PROs in all treatment cohorts, whereas MFIS5 (fatigue) was only answered in the METU cohort. Disability (PDDS) was among the top five reported PROs in the HETU and OU treatment cohorts. PDDS was not in the top five completed PROs in the METU cohort. As shown in Figure 2B, OUs on average answered respective PROs around 2 times during the entire observation period, whereas the average reporting for HETUs and METUs was lower, 1.7 times and 1.4 times, respectively. In the differentiation by PPMS and RMS, IVIS5 and PDQ-5 were only reported in the RMS group, whereas bladder control (BLCS) and fatigue (MFIS5) were reported only in the PPMS cohort (Figure 2C). 

Specifically, in the OU cohort, depression (BDI-FS) was only completed as one of the top five questionnaires in the youngest age group (18–25), whereas fatigue (MFIS-5) was one of the most answered PROs within the 26–35-year-old group. The age group 26–35 was the only age group to not report PES in the top five PROs. Disability (PDDS) was in the top five reported PROs for age groups over 36 years (Table 6).

### 3.4. Baseline PRO Scores—All Patients vs. Ocrelizumab Users

**Figure 3:**
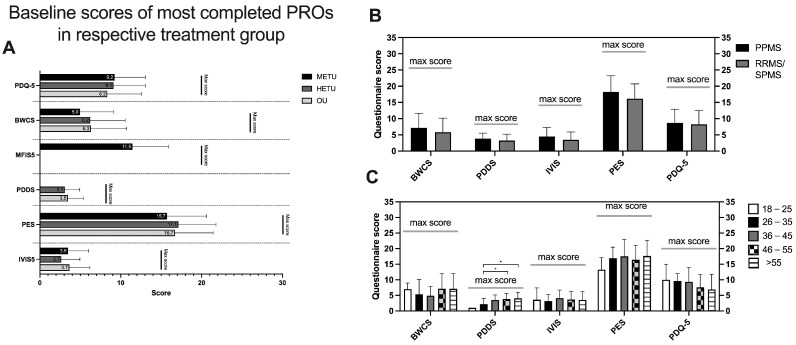
Baseline scores of top 5 completed PROs in respective treatment groups. (**A**) Brisa users were grouped into three categories based on the medications they use. Baseline scores of the top 5 most reported PROs in the respective medication group are depicted for ‘moderate-efficacy treatment users’ (METUs), ‘high-efficacy treatment users’(HETUs) and ‘ocrelizumab users (OUs)’. (**B**) Baseline scores of most reported PROs of ocrelizumab users grouped by MS type and (**C**) age group. Significant differences in base scores were calculated using multiple-comparison Kruskal–Wallis test correcting for *p*-values using Dunn’s test. * *p* < 0.05.

In addition, we assessed whether the baseline scores of the top five completed PROs vary depending on the medication class the Brisa users are treated with. Overall, the baseline scores between the treatment cohorts were similar (Figure 3A). Specifically, the average scores for bowel control (BWCS) and vision (IVIS5) were low, whereas scores for cognition (PDQ-5), fatigue (MFIS5), disability (PDDS) and pain (PES) were in the mid-range.

When OUs were analyzed in more detail, there were no significant differences in reported baseline scores between OUs diagnosed with PPMS and RRMS/SPMS users (Figure 3B), but the disability (PDDS score) increases with increasing age; specifically, age groups 46–55 and over 55 have significantly higher scores than the age group 26–35 (Figure 3C). 

## 4. Discussion

While clinical trial data have delivered an indispensable understanding of the efficiency and outcomes of disease-modifying treatments, data regarding day-to-day disease management based on RWE are still lacking. Although there are many MS companion apps on the market focusing on various use cases like medication tracking, symptom tracking or lifestyle tracking, Brisa is a first-of-its-kind medical device smartphone application in Germany intended not only to support MS patients in their day-to-day lives, but also to collect patient-centered data and experiences. 

Our retrospective analysis focused on the well-being of Brisa users on medium- and high-efficacy drugs, with a focus on ocrelizumab patients, and aimed to generate insights to narrow the gap between experimental research, clinical studies and real-world data of MS patients. 

Twenty-five percent of Brisa users declared the usage of ocrelizumab as their treatment. The characteristics of the ocrelizumab Brisa cohort are comparable to the findings of previous clinical trials [21,22] and non-interventional studies such as the CONFIDENCE study with more than 3000 participants [17]. With a mean age of 41.9 ± 11.2 years, our cohort was around 10 years younger than the participants of the CONFIDENCE study (51.3 ± 10.0 years) [23] and 5.6 years younger than the average age reported by the MS registry (47.5 ± 12.5 years) [24]. Recent studies show that younger users have a significantly higher affinity for using mobile technology in disease management [25] when compared to older users, which could explain why the average age of users was lower than in the registry and study data. The mean time since diagnosis in our cohort was 9.1 ± 7.2 years, whereas the mean time since diagnosis of the CONFIDENCE study participants was 5.5 ± 6.7 years. Our cohort was representative of the gender distribution (75% female) which is also reported by the MS registry in Germany, where 70.9% of MS patients are female. In our cohort, the share of RRMS/SPMS users decreased with age, whereas PPMS increased with age. This inverse relationship is also reflected in current data, where the median age of patients diagnosed with PPMS was 50 years, whereas RRMS patients are usually diagnosed in their 20s to 30s [26]. Overall, our cohort is representative of the described MS population in Germany, which allows us to extrapolate our findings to the overall MS population in Germany.

Our ocrelizumab cohort consisted of 73% users diagnosed with RRMS/SPMS and 27% diagnosed with PPMS. Symptoms of concern depend on MS type, which becomes apparent when analyzing the top five reported symptoms in the ocrelizumab cohort by MS type. According to the National MS Society, patients diagnosed with PPMS tend to have more lesions in the spinal cord than in the brain and therefore tend to experience more problems walking [27]. PPMS-diagnosed users are concerned with symptoms that affect the disability aspect, like spasticity cramps and leg foot lifting disorder, whereas RRMS/SPMS-diagnosed users are more concerned about the sensory spectrum of symptoms like tingling and pain. As ocrelizumab is the only highly effective treatment approved for PPMS, no PPMS-specific symptoms are reported in the top five in the HETU or METU group. 

PDDS was only in the top five answered PROs in the OU and HETU groups, but not the METU cohort. Our patients in the OU and HETU groups were older than those in the METU group, which could be one explanation as to why only these two groups answered PDDS questionnaires under the most common PROs. Overall, we would have expected PDDS to be under the most tracked PROs since it is an established way to determine the patient’s disease progression regarding disability. The voluntary aspect of answering the chosen PRO may be the reason other PROs were answered more frequently. 

Within the OU cohort, the average disability (PDDS) score in our PPMS cohort using ocrelizumab was 3.8, which is slightly lower than the score reported in the study on the real-world safety and effectiveness of ocrelizumab in patients with PPMS [23] which showed that the majority of patients had a significant disability at baseline (EDSS ≥ 4.0). The inclusion of PPMS patients, who experience more effects on mobility during their disease course, may be one additional explanation for the higher disability (PDDS) scores with increasing age in the OU cohort. 

In the OU cohort, both the disability (PDDS) and pain (PES) scores increased also with increasing age. Similarly, the symptom ‘leg foot lifting disorder’ concerns mainly the older users in the ocrelizumab cohort. This is most likely linked to a potential progression independent of relapses (PIRA), where the severity of disability increases over time [28].

Fatigue is one of the most common symptoms of MS, affecting about 80% of people [29]. In our cohort, across all treatment groups, the quick-check symptom fatigue is also tracked by the largest proportion of users. Interestingly, when comparing to the most reported PROs in the respective treatment cohort, MFIS-5 (fatigue) is only completed by around 22% of METUs, whereas fatigue PRO (MFIS-5) is not in the top five completed PROs in the OU and HETU treatment groups. This could be due to several reasons. Symptom tracking in the Brisa app occurs daily, whereas the PROs are completed every 2 weeks. It is possible that since fatigue is such a common symptom and affects most MS patients, users do not see the need to track the PROs in addition to the daily symptom quick check. Instead, users focus on the disease symptoms that are underrepresented in the daily symptom check.

Although depression in its various forms is one of the most common symptoms of MS [29], it is striking that young users aged 18–25 years tracked this symptom more often than older age groups. One explanation could be that younger people are more aware of psychological issues, but more research is needed to understand the reasons behind this. 

A follow-up analysis comparing the scores over time will give greater insight into the ability to track disease development using a companion app. We could not identify any significant differences in baseline PRO scores for disability (PDQ-5), bowel control (BWCS), pain (PES) and vision (IVIS5) between the three treatment cohorts.

Overall, both the daily symptom quick check and the PROs are completed more often in the OU treatment group compared to the other treatment groups. One hypothesis is that the patients on a high-efficacy treatment are perceiving a measurable improvement and want to document their therapy success, although this hypothesis would need to be verified in a follow-up study to measure the perceived outcomes of patient treatments.

Analyses involving patient-reported data entail the probability of a small percentage of false data inputs by users themselves, which cannot be overlooked. Also, the lack of additional information, especially information regarding the frequency of flare-ups, periods of remission, other types of medications used, etc., limited us from gathering further insights and drawing associations between symptoms and medications. With the continuation of the application development, these features could potentially contribute to a fuller picture of patient-reported data.

## 5. Conclusions

In conclusion, the methodology of collecting patient-centered data in a chronic disease such as MS is essential for improving patient care and treatment outcomes and provides an angle that is fundamental for patient-centered treatment approaches. Digital companion apps, such as Brisa, provide a means for the collection of real-time data on a longitudinal basis, which is crucial to our understanding of MS pathophysiology and progression, and serve as tools for communication between patients and physicians. Studies have shown that patient involvement is crucial for the development of a meaningful patient companion in an iterative way [30]. 

The current study’s findings could potentially lead to a better understanding of MS and improved patient care for those on ocrelizumab treatment. However, these findings raise new questions, such as what the increased reporting willingness is due to and what the underlying reasons for patients’ reporting behavior are. Answering these questions requires further research and analysis.

## Figures and Tables

**Table 1:** Medication grouping.

Moderate-Efficacy Treatment Users (METUs)	High-Efficacy Treatment Users (HETUs)	Ocrelizumab Users (OUs)
Dimethylfumarate	Alemtuzumab	Ocrelizumab
Diroximelfumarate	Cladribine	
Glatirameracetate	Natalizumab	
Teriflunomide	Ofatumumab	
Interferons	S1P Modulators (Fingolimod, Ozanimod, Ponesimod)	

**Table 2:** Demographic characteristics of Brisa users. Distribution of Brisa users (*n* = 1593) taking ocrelizumab (*n* = 405) compared to non-ocrelizumab users (*n* = 1188) and distribution of Brisa users regarding gender and MS type.

Brisa Users (*n* = 1593)	Non-Ocrelizumab Users (*n* = 1188)			Ocrelizumab Users (*n* = 405)		
Gender	Female	Male	Divers	Female	Male	Divers
	1021	167	0	306	98	1
MS Type	SP	PP	RR	SP	PP	RR
	53	0	1135	42	108	255

**Table 3:** Age and time since diagnosis distribution of Brisa users as total number (*n*) and % of users within medication group.

	OUs (*n* = 405)	HETUs (*n* = 884)	METUs (*n* = 709)
Age group			
18–25	24 (5.9%)	68 (7.7%)	57 (8.0%)
26–35	113 (27.9%)	275(31.1%)	241 (34.0%)
36–45	105 (25.9%)	251 (28.4%)	207 (39.2%)
46–55	105 (25.9%)	204 (23.1%)	143 (20.2%)
>55	58 (14.3%)	86 (9.7%)	61 (8.6%)
Time since diagnosis			
0–1	9 (2.2%)	28 (3.2%)	45 (6.3%)
2–5	142 (35.1%)	280 (31.7%)	286 (40.3%)
6–10	108 (26.7%)	246 (27.8%)	176 (24.8%)
11–20	91 (22.5%)	225 (25.5%)	128 (18.1%)
21–30	55 (13.6%)	105 (11.9%)	74 (10.4%)

**Table 4:** Age and time since diagnosis distribution of ocrelizumab users as total number (*n*) and % of users within MS Type.

	RRMS	PPMS
Age group		
18–25	22 (7.4%)	2 (1.9%)
26–35	104 (35.0%)	9 (8.3%)
36–45	81(27.3%)	24 (22.2%)
46–55	64 (21.5%)	41(38.0%)
>55	26 (8.8%)	32 (29.6%)
Time since diagnosis		
0–1	6 (2.0%)	3 (2.8%)
2–5	112 (37.7%)	56 (51.9%)
6–10	75 (25.3%)	24 (22.2)
11–20	64 (21.5%)	15 (13.9)
21–30	40 (13.5%)	10 (9.3)

**Table 5:** Top 5 reported symptoms within OU group based on age group.

Age Group										
Symptom	18–25 (*n* = 23)		26–35 (*n* = 103)		36–45 (*n* = 96)		46–55 (*n* = 98)		>56 (*n* = 48)	
	*n*	% of Age Group	*n*	% of Age Group	*n*	% of Age Group	*n*	% of Age Group	*n*	% of Age Group
Fatigue	10	43.5	26	25.2	34	35.4	33	33.7	17	35.4
Numbness	7	30.4								
Pain	7	30.4	21	20.4	23	24.0	26	26.5		
Bladder disorder	5	21.7							12	25.0
Depression	5	21.4								
Tingling			22	21.4			28	28.6	12	25.0
Concentration disorder			29	28.2	24	25.0				
Visual disturbances			20	19.4						
Strong sensitivity to heat					28	29.2				
Spasticity/cramps					25	26.0	26	26.5	14	29.2
Leg foot lifting disorder							25	25.5	15	31.3

**Table 6:** Top 5 completed PROs within OU group based on age group.

Age Group										
PRO	18–25 (*n* = 18)		26–35 (*n* = 79)		36–45 (*n* = 73)		46–55 (*n* = 76)		>56 (*n* = 48)	
	*n*	% of Age Group	*n*	% of Age Group	*n*	% of Age Group	*n*	% of Age Group	*n*	% of Age Group
PES (pain)	8	44.4	16	20.3	26	35.6	30	39.5	12	25.0
IVIS (vision)	5	27.8	28	35.4	33	45.2	21	27.6	11	22.9
BDI-FS (depression)	4	22.2								
PDQ-5 (Cognition)	4	22.2	17	21.5	21	28.8	20	26.3		
BLWS (bladder control)	3	16.7								
BWCS (bowel control			19	24.1					18	37.5
MF5I (fatigue)			16	20.3	20	27.4	17	22.4	10	20.8
PDDS (disability)					24	32.9	29	38.2	21	43.8

## Data Availability

The data are not publicly available due to data protections regulations.

## Supplementary Materials

The following supporting information can be downloaded at: https://www.mdpi.com/article/10.3390/jpm14040409/s1, Ref. [31] is cited in Supplementary Materials File. Figure S1: Smiley-face based rating system used in Brisa app. Figure S2: Distribution of medications among Brisa users.

## Abbreviations

BDI-FS	Beck Depression Inventory—Fast Screen
BLCS	Bladder Control Scale
BWCS	Bowel Control Scale
DMT	disease-modifying therapy
EBV	Epstein–Barr virus
GDPR	General Data Protection Regulation
IVIS	Impact of Visual Impairment Scale
MFIS-5	Modified Fatigue Impact Scale—5-Item Version
MS	multiple sclerosis
PDDS	Patient-Determined Disease Steps
PDQ-5	Perceived Deficits Questionnaire—5-Item Version
PRO	patient-reported outcome
PES	MOS Pain Effects Scale
PPMS	primary progressive MS
RRMS	relapsing–remitting MS
RWE	real-world evidence
SPMS	secondary progressive MS
SSS	Sexual Satisfaction Scale
