# Supplementary material for: Data on Ocrelizumab Treatment Collected by MS Patients in Germany Using Brisa App
Source: J Pers Med. 2024 Apr 12;14(4):409. doi: 10.3390/jpm14040409 (PMC11051290; doi:10.3390/jpm14040409)
Supplement: Supplementary file 1 [file jpm-14-00409-s001.zip › jpm-2952904-supplementary.pdf]

Patient collected data on ocrelizumab treatment by MS patients in Germany using Brisa app

Supplement

Supplemental Methods: Detailed description of all PRO questionnaires used in Brisa app.

Beck Depression Inventory - Fast Screen (BDI-FS)

The Beck Depression Inventory - Fast Screen is derived from Beck Depression Inventory II for measuring the severity level of depression in patients with possible physical limitations. The questionnaire consists of 7 groups of statements. For each statement there are four possible answers, which are ordered according to their intensity and assigned a score from 0–3. The individual scores are summed up and can result in a total score of 0–21, with a higher score indicating greater severity of depression.

Bladder Control Scale (BLCS)

The Bladder Control Scale (BLCS) was used to measure bladder problems that may occur in MS. The answering of the question refers to the last 4 weeks before answering the questionnaire. It is a set of 4 items to measure bladder control and the extent to which bladder problems have an impact on everyday activities. The first 3 items are rated on a scale of 0–4, where 0 indicates "not at all" and 4 indicates "daily". The fourth item is rated in a scale of 0–10, where 0 indicates "not at all" and 10 indicates "severely". Each participant receives a total score between 0 and 22, with higher scores representing poorer bladder control and a greater impact of this on daily activities.

Bowel Control Scale (BWCS)

Bowl problems that may occur in MS and their impact on everyday activities was measured with the Bowel Control Scale (BWCS). It is a set of 5 items to measure bowl control and the extent to which bowl problems have an impact on daily activities. The first 4 items are rated on a scale of 0–4, where 0 indicates "not at all" and 4 indicates "daily". The fifth item is rated in a scale of 0–10, where 0 indicates "not at all" and 10 indicates "severely". Each participant receives a total score between 0 and 26, with higher scores indicating poorer bowel control and stronger impairment of daily activities.

Patient-determined Disease Steps (PDDS)

To visualize the progression of MS in patients, the Patient-determined Disease Steps questionnaire was used. This consists of a list of 9 categories describing disease severity with a special focus on the mobility of MS patients. Each of the 9 categories is assigned a score from 0–8. The patient selects the appropriate category and receives the corresponding score. A higher score describes a reduced mobility [28].

Modified Fatigue Impact Scale – 5-Item Version (MFIS-5)

The Modified Fatigue Impact Scale - 5-Item Version was used to measure how fatigue may affect patients. The answering of the question refers to the last 4 weeks before answering the questionnaire. It is a set of 5 items to measure fatigue and the extent to which fatigue has an impact on everyday activities. The 5 items are rated on a scale of 0–4, where 0 indicates "never" and 4 indicates "almost always". Each participant receives a total score between 0 and 20, with higher scores representing greater severity of fatigue and greater impairment of daily activities.

Impact of Visual Impairment Scale (IVIS)

The Impact of Visual Impairment Scale was used to assess visual problems that can occur in MS and their impact on daily activities. It is a series of 5 questions about visual impairments and the extent to which they affect daily activities. The questions are scored on a scale of 0 to 3, with 0 being 'not at all difficult' and 3 being 'not possible due to visual problems'. Each participant is given a total score between 0 and 15, with higher scores indicating greater impairment of vision and therefore greater impairment of daily living.

MOS Pain Effects Scale (PES)

The MOS Pain Effects Scale was used to measure the experience of unpleasant sensory symptoms because of MS. The answering of the question refers to the last 4 weeks before answering the questionnaire. It is a set of 6 items to measure how much the sensory symptoms interfere with daily life. The 6 items are rated on a scale of 1–5, where 1 indicates "not at all" and 5 indicates "to an extreme degree". Each participant receives a total score between 6 and 35, with higher scores representing greater interfering of the unpleasant sensory symptoms with everyday activities.

Perceived Deficits Questionnaire - 5-Item Version (PDQ-5)

The Perceived Deficits Questionnaire - 5-Item Version (PDQ-5) was used to assess cognitive dysfunction specific to MS. It is a series of 5 items scored on a scale of 0 to 4, with 0 representing "never" and 4 representing "almost always", to measure different areas of cognitive function that are commonly impaired in MS. The total score ranges from 0 to 20, with higher scores representing more pronounced cognitive dysfunction.

Sexual Satisfaction Scale (SSS)

To assess the impairment of sexual function in patients with MS, we have used the Sexual Satisfaction Scale. The questionnaire consists of a total of 5 questions, whereby the first question can only be answered with yes/no with a corresponding score of 0–1. The other questions are scored from 1–6, where 1 stands for "very satisfied" and 6 for "very dissatisfied". The total score can be between 0–25. A higher score indicates more pronounced sexual dissatisfaction.

Supplemental Methods: Detailed description of all symptoms used in Brisa app.

Users can track their symptoms daily by answering their symptom severity on a smiley-face based rating system. As shown in Supplemental Fig. 1, symptoms were rated using 5 different smileys ranging from "very sad" to "very happy". These smileys were assigned a score of 0-4, with "very sad" being given a score of 0 and "very happy" a score of 4.

Supplemental Figure S1: Smiley-face based rating system used in Brisa app.

Among others, the symptoms "depression", "bladder disorders", "bowel disorders", "leg-foot-lifting disorders", "fatigue", "visual disturbances", "pain", "cognitive disorders", "concentration disorders", "forgetfulness" and "sexual dysfunction" were considered. To identify the symptoms that predominantly affected our study cohort, users who tracked at least 1 symptom, once after onboarding were considered. The total number of users who answered each symptom was calculated and the top 5 symptoms were identified.

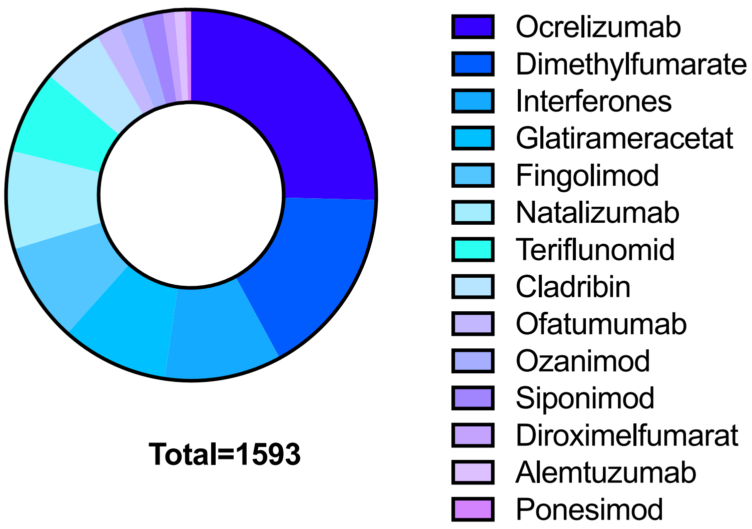

Supplemental Figure S2: Distribution of medications among Brisa users

Supplemental References

[31] Learmonth YC, Motl RW, Sandroff BM, Pula JH, Cadavid D. Validation of patient determined disease steps (PDDS) scale scores in persons with multiple sclerosis. BMC Neurol 2013;13:37. https://doi.org/10.1186/1471-2377-13-37.
